# Dual regulation of cytosolic ascorbate peroxidase (APX) by tyrosine nitration and *S*-nitrosylation

**DOI:** 10.1093/jxb/ert396

**Published:** 2013-11-28

**Authors:** Juan C. Begara-Morales, Beatriz Sánchez-Calvo, Mounira Chaki, Raquel Valderrama, Capilla Mata-Pérez, Javier López-Jaramillo, María N. Padilla, Alfonso Carreras, Francisco J. Corpas, Juan B. Barroso

**Affiliations:** ^1^Área de Bioquímica y Biología Molecular, Departamento de Biología Experimental, Facultad de Ciencias Experimentales, Ed. B3. Campus Universitario “Las Lagunillas” s/n, Universidad de Jaén, E-23071 Jaén, Spain; ^2^Instituto de Biotecnología, Universidad de Granada, Spain; ^3^Departamento de Bioquímica, Biología Celular y Molecular de Plantas, Estación Experimental del Zaidín (EEZ), Consejo Superior de Investigaciones Científicas, E-18080 Granada, Spain

**Keywords:** Ascorbate peroxidase, nitration, nitric oxide, *S*-nitrosoglutathione, *S*-nitrosylation, peroxynitrite, reactive nitrogen species, salinity stress.

## Abstract

Post-translational modifications (PTMs) mediated by nitric oxide (NO)-derived molecules have become a new area of research, as they can modulate the function of target proteins. Proteomic data have shown that ascorbate peroxidase (APX) is one of the potential targets of PTMs mediated by NO-derived molecules. Using recombinant pea cytosolic APX, the impact of peroxynitrite (ONOO^–^) and *S*-nitrosoglutathione (GSNO), which are known to mediate protein nitration and *S*-nitrosylation processes, respectively, was analysed. While peroxynitrite inhibits APX activity, GSNO enhances its enzymatic activity. Mass spectrometric analysis of the nitrated APX enabled the determination that Tyr5 and Tyr235 were exclusively nitrated to 3-nitrotyrosine by peroxynitrite. Residue Cys32 was identified by the biotin switch method as *S*-nitrosylated. The location of these residues on the structure of pea APX reveals that Tyr235 is found at the bottom of the pocket where the haem group is enclosed, whereas Cys32 is at the ascorbate binding site. Pea plants grown under saline (150mM NaCl) stress showed an enhancement of both APX activity and *S*-nitrosylated APX, as well as an increase of H_2_O_2_, NO, and *S*-nitrosothiol (SNO) content that can justify the induction of the APX activity. The results provide new insight into the molecular mechanism of the regulation of APX which can be both inactivated by irreversible nitration and activated by reversible *S*-nitrosylation.

## Introduction

The discovery of nitric oxide (NO) generation in cells and related molecules known as reactive nitrogen species (RNS) has shown that these molecules can mediate additional post-translational modifications (PTMs) such as nitration and *S*-nitrosylation. Protein tyrosine nitration adds a nitro group (-NO_2_) to one of the two equivalent ortho-carbons of the tyrosine residue aromatic ring. This converts tyrosine into a negatively charged hydrophilic nitrotyrosine moiety and causes a marked shift in the local p*K*_a_ of the hydroxyl group from 10.07 in tyrosine to 7.50 in nitrotyrosine (Turko and Murad, 2002). This PTM is regarded as a process which depends on factors such as protein structure, the nitration mechanism, and the environmental compartments where the protein is located. These covalent changes may result in effects such as loss or gain in protein function or no change in function (Souza *et al.*, 2008; Radi, 2013). *S*-nitrosylation consists of binding an NO group to a protein cysteine residue and can also change the function of many proteins. Through proteomics analysis, a significant number of plant protein target candidates for *S*-nitrosylation have been identified, including the cytoskeleton, metabolic, redox-related, stress-related and signalling/regulating proteins. However, up to now, only a limited number of proteins have been studied in order to determine how they are regulated by this PTM at the molecular level (Astier *et al.*, 2012; Begara-Morales *et al.*, 2013*b*).

Ascorbate peroxidase (APX) together with glutathione reductase, monodehydroascorbate reductase, and dehydroascorbate reductase plus the antioxidant metabolites ascorbate, glutathione, and NADPH constitute the ascorbate–glutathione cycle. This metabolic pathway is essential for the detoxification and regulation of the cellular level of hydrogen peroxide (H_2_O_2_) (Asada, 1992; Noctor and Foyer, 1998). APX catalyses the electron transfer from ascorbate to H_2_O_2_, thus giving rise to dehydroascorbate and water as products. This enzyme has been identified in many higher plants and comprises a family of isoenzymes with distinct characteristics which are distributed throughout the different cell compartments including the cytosol, chloroplasts, peroxisomes, and mitochondria (for a review, see Shigeoka *et al.*, 2002). In higher plants, APX is an essential element in the fine-tuning regulation mechanism of H_2_O_2_ during plant development and under different environmental stresses. Consequently, APX has been analysed in terms of both physiological and biochemical aspects such as catalytic regulation, enzyme–ligand interactions, molecular properties, structure, subcellular localization, gene regulation, and responses to biotic and abiotic stress (Nakano and Asada, 1981; Bunkelmann and Trelease, 1996; Corpas and Trelease, 1998; Karpinski *et al.*, 1997; Jiménez *et al.*, 1998; Yoshimura *et al.*, 1999, 2000; Rossel *et al*., 2002; Wada *et al.*, 2003; Sharp *et al.*, 2003; Koussevitzky *et al.*, 2008).

Recently, APX has been identified as a potential target of tyrosine nitration in *Arabidopsis* (Lozano-Juste *et al.*, 2011) and *Citrus aurantium* (Tanou *et al.*, 2012), and NO has been shown to be capable of modulating its activity in different ways through either inactivation (Clark *et al.*, 2000) or activation (Keyster *et al.*, 2011; Lin *et al.*, 2011). Proteomic analysis has also identified APX as a target of *S*-nitrosylation in *Arabidopsis* plants (Fares *et al.*, 2011). With the aim of determining which mechanism(s) is(are) involved in the modulation of APX by NO-derived species, an initial pharmacological analysis using recombinant pea cytosolic APX was carried out. Furthermore, the analysis of APX under salinity stress also supports that the *S*-nitrosylation of APX contributed in the mechanism of response against the nitro-oxidative stress provoked by salinity in pea plants. Data enabled the demonstration that pea APX is modulated by both irreversible tyrosine nitration and reversible *S*-nitrosylation which lead to antagonistic effects: nitration of Tyr235 inhibits APX activity while *S*-nitrosylation of Cys32 causes an increase in APX activity, indicating an interplay between NO metabolism and a relevant antioxidant enzyme involved in ROS metabolism.

## Materials and methods

### Plant material and growth conditions

Pea (*Pisum sativum* L., cv. Lincoln) seeds were obtained from Royal Sluis (Enkhuizen, The Netherlands). Seeds were surface sterilized with 3% (v/v) commercial bleaching solution for 3min, and then were washed with distilled water, and germinated in vermiculite for 3–4 d under the following growth chamber conditions: 24 ºC/18 ºC (day/night), 80% relative humidity, a 16h photoperiod, and a light intensity of 190 µE m^–2^ s^–1^. Healthy and vigorous old seedlings were selected and grown in a nutrient solutions (Corpas *et al.*, 1993). After 14 d, plants were transplanted to similar media supplemented with 150mM NaCl and were grown for 4 d.

### Crude extract of pea leaves

Leaves from control and NaCl-treated pea plants were ground in liquid nitrogen using a mortar and pestle. The resulting powder was added to 1/3 (w/v) extraction medium of 25mM HEPES buffer, pH 8.0, containing 1mM diethylenetriaminepentaacetic acid (DTPA) and 0.1mM neocuproine. The crude extracts were then filtered through one layer of Miracloth (Calbiochem, San Diego, CA, USA), centrifuged at 3000 *g* for 6min (4 ºC), and the supernatants were used for the *S*-nitrosylated protein analysis by the biotin switch method.

### Expression and purification of cytosolic pea APX

**Fig. 1.**
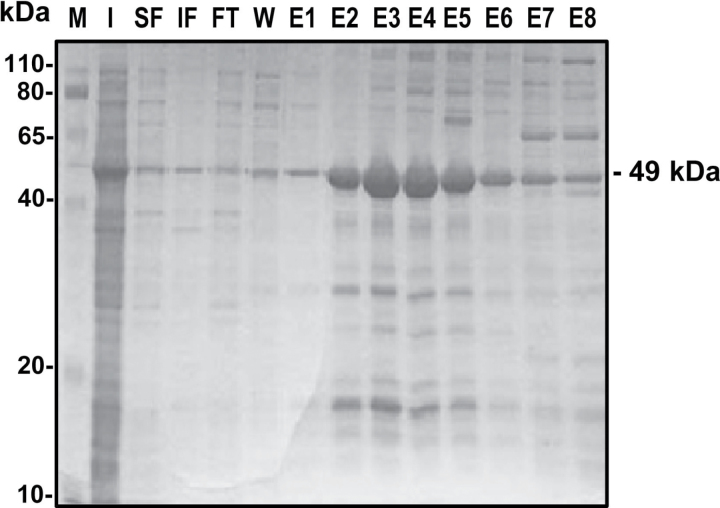
SDS–PAGE analysis of the purification of the recombinant cytosolic ascorbate peroxidase (APX). The gel was stained with Coomasie blue. M, molecular markers; I, total protein in induced culture; SF, soluble fraction; IF, insoluble fraction; FT, flow-through; W, wash; E1–E8, elution fractions.

The cDNA encoding mature pea cytosolic APX (M93051) was amplified by PCR from total pea leaf RNA using the Fast Start High Fidelity polymerase (Roche) and the specific primer sets: 5′-GGATCCTATGGGAAAATCATACCCAACTG-3′ and 5′-CTC GAGTCTTAGGCTTCAGCAAATCCAAG-3′. The PCR product (766bp) was cloned into the pGEM-T Easy Vector (Promega). The positive clones were confirmed by sequencing and then subcloned prior to digestion with *Bam*HI and *Xho*I into the pALEXb vector. Recombinant protein carrying an N-terminal choline-binding domain was produced using *Escherichia coli* strain BIVU0811, which was routinely cultured overnight at 37 ºC in LB kanamycin (25mg l^–1^) and ampicillin (100mg l^–1^). Gene expression was induced by the addition of 1mM salicylate and 10mM 3-methyl benzoate in 250ml of culture grown at 20 ºC overnight in order to produce a higher proportion of soluble protein. Cells were harvested by centrifugation and resuspended in 20ml of phosphate-buffered saline (PBS) (pH 7.0) containing 25U ml^–1^ DNase I, 10mM MgCl_2_, and commercial protease inhibitor (Complete, Roche). Cells were lysed with a Niro Soavi NS1001L Panda High-Pressure homogenizer at a pressure of 800–900 bar. The cell lysate was then centrifuged at 10 000 *g* at 4 ºC for 15min, and the supernatant was used for the purification of recombinant protein using a 1ml LYTRAP column (Biomedal). The column was washed with 20ml of 20mM K phosphate buffer (pH 7.0) containing 300mM NaCl and 5mM choline. The protein was eluted in 1ml fractions using a discontinuous gradient of choline prepared in the same buffer with 100mM NaCl and 20mM choline (fraction E1), 50mM choline (E2), 75mM choline (E3), 100mM choline (E4), 150mM choline (E5), 200mM choline (E6), 250mM choline (E7), and 500mM choline (E8). The samples were analysed by 10% SDS–PAGE and stained with Coomassie (Fig. 1).

### Ascorbate peroxidase activity assay. Treatment with SIN-1 (peroxynitrite donor) and GSNO (nitric oxide donor)

APX (EC 1.11.1.11) activity was determined by monitoring the initial ascorbate oxidation by H_2_O_2_ at 290nm (Hossain and Asada, 1984). The molecule SIN-1 (3-morpholinosydnonimine) has been shown to generate peroxynitrite, a protein-nitrating compound (Daiber *et al.*, 2004). Cytosolic recombinant APX was therefore incubated at 37 ºC for 1h with increasing concentrations (0–5mM) of SIN-1 (Calbiochem) freshly made up before use. For treatments with NO donors, cytosolic recombinant APX was incubated at room temperature for 30min with 0.5mM and 2mM GSNO. As a control, the sample was also incubated with 0.5mM and 2mM glutathione (GSH). The protein concentration was determined with the aid of the Bio-Rad Protein Assay using bovine serum albumin (BSA) as standard.

### Identification of nitrated tyrosine in recombinant cytosolic pea APX using mass spectrometric techniques (LC-MS/MS)

Purified recombinant pea cytosolic APX was processed according to a protocol involving reduction with dithiothreitol (DTT), derivatization with iodoacetamide (IAA), and enzymatic digestion with trypsin (37 ºC, 8h). The sample was purified using solid-phase extraction cartridges to eliminate choline interference. The resulting peptide mixture was analysed using a MALDI-TOF/TOF mass spectrometer (4800, AB Sciex) to evaluate the quality of the sample. MALDI-TOF spectra were interpreted using a peptide mass fingerprinting (PMF) database search (Protein Prospector program). The database used for identification was UniProt (release 2011_02). The sample was then analysed by liquid chromatography–tandem mass spectrometry (LC-MS/MS) using a Velos-LTQ mass spectrometer equipped with a micro-ESI (electrospray ionization) ion source (ThermoFisher, San Jose, CA, USA). The sample was evaporated to dryness and diluted up to 40 μl with water containing 5% methanol and 1% formic acid. The sample was then loaded in a chromatographic system consisting of a C18 pre-concentration cartridge (Agilent Technologies, Santa Clara, CA, USA) connected to a 10cm long, 150 μm i.d. Vydac C18 column (Vydac, IL, USA). The separation was carried out at 1 μl min^–1^ with a 3–40% acetonitrile gradient for 30min (solvent A, 0.1% formic acid; solvent B, acetonitrile with 0.1% formic acid). The HPLC system contained an Agilent 1200 capillary pump, a binary pump, a thermostated microinjector, and a micro switch valve.

The Velos-LTQ instrument was operated in positive ion mode with a spray voltage of 2kV. The scan range of each full MS scan was *m/z* 400–2000. After each MS scan, a collection of targeted MS/MS spectra was obtained in order to identify both the unmodified and nitrated form of the predicted tyrosine-containing peptides. The parent mass list of the targeted scan was selected to ensure maximum coverage of the tyrosine-containing tryptic peptides for APX. The list of targeted *m/z* values was obtained after *in silico* digestion of the proteins using nitrated tyrosine as a dynamic modification. The resulting list of predicted peptides (in both nitrated and unmodified form) was filtered to exclude all peptides not containing tyrosine residues.

MS/MS spectra were searched using Proteome Discoverer software (ThermoFisher) on the basis of the following parameters: peptide mass tolerance 2Da, fragment tolerance 0.8Da, enzyme set as trypsin, and no missed cleavages. The dynamic modifications were cysteine carbamidomethylation (+57Da), methionine oxidation (+16Da), and tyrosine nitration (+45). The searches were carried out using a database containing all the proteins listed in Table 1. Identifications were filtered with XCorr >3, P(pep) <15%. The MS/MS spectra of the nitrated tyrosines were manually validated by comparing the spectra obtained for the unmodified peptide and the nitrated peptide.

**Table 1.** List of peptides scanned and peptides identified by LC-MS/MS

Peptides identified^*a*^	Peptides scanned	Length (amino acids)	Mr (Da)		No. of tyrosines
			Not nitrated	Nitrated	
	EQFPIVSYADFYQLAGVVAVEITGGPEVPFHPGR	34	3691	–	2
DVFGKAMGLSDQDIVALSGGHTIGAAHKER		30	3082	–	0
SGFEGPWTSNPLIFDNSYFTELLTGEK	SGFEGPWTSNPLIFDNSYFTELLTGEK	27	3050	–	1
SYPTVSPDYQK	SYPTVSPDYQK	11	1284	1329	2
YAADEDVFFADYAEAHLK	YAADEDVFFADYAEAHLK	18	2074	2119	2

Some of the detected peptides do not contain tyrosines. These peptides were not included in the targeted MS/MS detection. They were detected and identified because their molecular weight coincides with that of expected peptides.

### Biotin switch method

For *in vitro S*-nitrosylation, recombinant APX was incubated with *S*-nitrosoglutathione (GSNO) for 30min at room temperature. *S*-nitrosylated APX and crude extracts obtained from control and NaCl-treated pea plants were subjected to the biotin switch method as described by Jaffrey *et al.* (2001) with some slight modifications. Blocking of the non-nitrosylated free cysteine residue was carried out by incubation with 30mM methyl methanethionsulphonate and 2.5% SDS at 50 °C for 20min with frequent vortexing. Residual methyl methanethionsulphonate was removed by precipitation with 2 vols of –20 °C acetone, and the proteins were resuspended in 0.1ml of HENS buffer (250mM HEPES pH 7.7 buffer containing 1mM EDTA, 0.1mM neocuproine, and 1% SDS) per mg protein. Biotinylation was achieved by adding 1mM *N*-[6-(biotinamido)hexyl]-3′-(2′-pyridyldithio) propionamide (biotin-HPDP) and 0.1mM ascorbate, and incubating at room temperature for 1h. Then proteins were precipitated with 2 vols of –20 ºC acetone. Biotin-labelled proteins were separated by non-reducing 10% SDS–PAGE, and transferred onto polyvinylidene difluoride (PVDF) membranes (Immobilon P, Millipore, Bedford, MA, USA) using a semi-dry transfer system (Bio-Rad Laboratories). PVDF membranes were blocked with TRIS-buffered saline (TBS)+1% BSA. The blot was incubated with anti-biotin antibody at a dilution of 1:200 000 for 1h, and immunoreactive bands were detected using a photographic film (Hyperfilm, Amersham Pharmacia Biotech) with an enhanced chemiluminescence kit (ECL-PLUS, Amersham Pharmacia Biotech).

### Molecular evolution studies and analysis of the structure

Molecular evolution studies were carried out at the Evolutionary Trace server (Mihalek *et al.*, 2004) using the model of the tertiary structure of the pea APX as input, and the evolutionary conservation was ranked according to the rho parameter that deviates from 1 as the variability increases. The accessible solvent area (ASA) was analysed with DSSP (Kabsch and Sande, 1983). Molecular graphics and analyses were performed with the UCSF Chimera package (Pettersen *et al.*, 2004). Alignments and phylogenic tree were performed using Clustal W2.1 of APX amino acid sequences found in the peroxidase database (http://peroxibase.toulouse.inra.fr/).

### Lipid peroxidation and H_2_O_2_ content

Lipid peroxidation was estimated by determining the concentration of malondialdehyde (MDA) with thiobarbituric acid (Buege and Aust, 1978). Hydrogen peroxide content was determined by a spectrofluorometric assay (Creissen *et al.*, 1999)

### Detection of nitric oxide (NO) and *S*-nitrosothiols (SNOs) by CLSM

NO was detected with 10 μM 4,5-diaminoflorescein diacetate (DAF-FM DA; Calbiochem) prepared in 10mM TRIS-HCl (pH 7.4). Leaf cross-sections were incubated at 25 °C for 1h, in darkness, according to Corpas *et al.* (2008). After incubation, samples were washed twice in the same buffer for 15min each. Then leaf sections were embedded in a mixture of 15% acrylamide/bisacrylamide stock solution as described elsewhere (Corpas *et al.*, 2008), and 80–100 μm thick sections, as indicated by the vibratome scale, were cut under 10mM PBS. Sections were then soaked in glycerol/PBS containing azide (1:1, v/v) and mounted in the same medium for examination by confocal laser scanning microscopy (CLSM; Leica TCS SL), using standard filters and collection modalities for DAF-2 green fluorescence (excitation 495nm; emission 515nm).

SNOs were detected using the fluorescent reagent Alexa Fluor 488 Hg-link phenylmercury (Molecular Probes, Eugene, OR, USA) according to Chaki *et al.* (2009). Briefly, leaf cross-sections of ~25mm^2^ were incubated at 25 °C for 1h, in darkness, with 10mM *N-*ethylmaleimide (NEM) prepared in ethanol, and then were washed three times in 10mM TRIS-HCl buffer, pH 7.4, for 15min each. Next, the leaf samples were incubated with 10 μM Alexa Fluor 488 Hg-link phenylmercury for 1h at 25 °C, in darkness. After washing three times in the previous buffer, leaf sections were embedded in a mixture of 15% acrylamide–bisacrylamide stock solution and were processed as described above. The pea leaf sections were analysed with a CLSM system using standard filters for Alexa Fluor 488 green fluorescence (excitation 495nm; emission 519nm).

### Purification of biotinylated proteins and APX immunodetection

Purification of biotinylated proteins from control and NaCl-treated pea plant leaves was carried out as described by Sell *et al.* (2008) with slight modifications. Biotinylated proteins and 30 μl of neutravidin agarose 50% (w/v) slurry (high capacity neutravidin agarose resin, Thermo Scientific) per milligram of protein were equilibrated with a neutralization buffer [10mM HEPES pH 7.7 containing 100mM NaCl, 1mM EDTA, and 0.5% (v/v) Triton X-100]. Proteins were added to the neutravidin agarose matrix and were incubated 1h at room temperature with gentle shaking. The matrix with bound proteins was washed several times with washing buffer [20mM HEPES pH 7.7 containing 600mM NaCl, 1mM EDTA, and 0.5% (v/v) Triton X-100] and was transferred to an empty column. Finally, biotinylated proteins were eluted after incubation for 30min with elution buffer (20mM HEPES pH 7.7 containing 0.1M NaCl, 1mM EDTA, and 100mM β-mercaptoethanol) at room temperature. Purified biotinylated proteins were separated by 12% SDS–PAGE and transferred to PVDF membranes as described above. For APX immunodetection, membrane was incubated with a rabbit polyclonal antibody against cucumber APX (Corpas and Trelease, 1998) diluted 1:3000. Immunoreactive bands were detected using a photographic film (Hyperfilm, Amersham Pharmacia Biotech) with an enhanced chemiluminescence kit (ECL-PLUS, Amersham Pharmacia Biotech).

## Results

### Expression and purification of cytosolic APX. Effect of peroxynitrite (ONOO^–^)

As a means to increase our knowledge of the regulation mechanism of pea APX, the recombinant protein was obtained by sequencing the pea clone and overexpression in *E. coli* (see the Materials and methods). Figure 1 shows the electrophoretic analysis of the different fractions obtained after LYTRAP affinity column chromatography of the recombinant APX. The recombinant APX showed a molecular mass of ~49kDa which is in range of the theoretical value predicted for the cytosolic APX protein (27.7kDa) with the Ly-tag (21.28kDa). The E6 fraction with an APX activity of 204 nmol ascorbate min^–1^ mg^–1^ protein showed an adequate purity grade for this protein and it was used for the subsequent experiments.

**Fig. 2.**
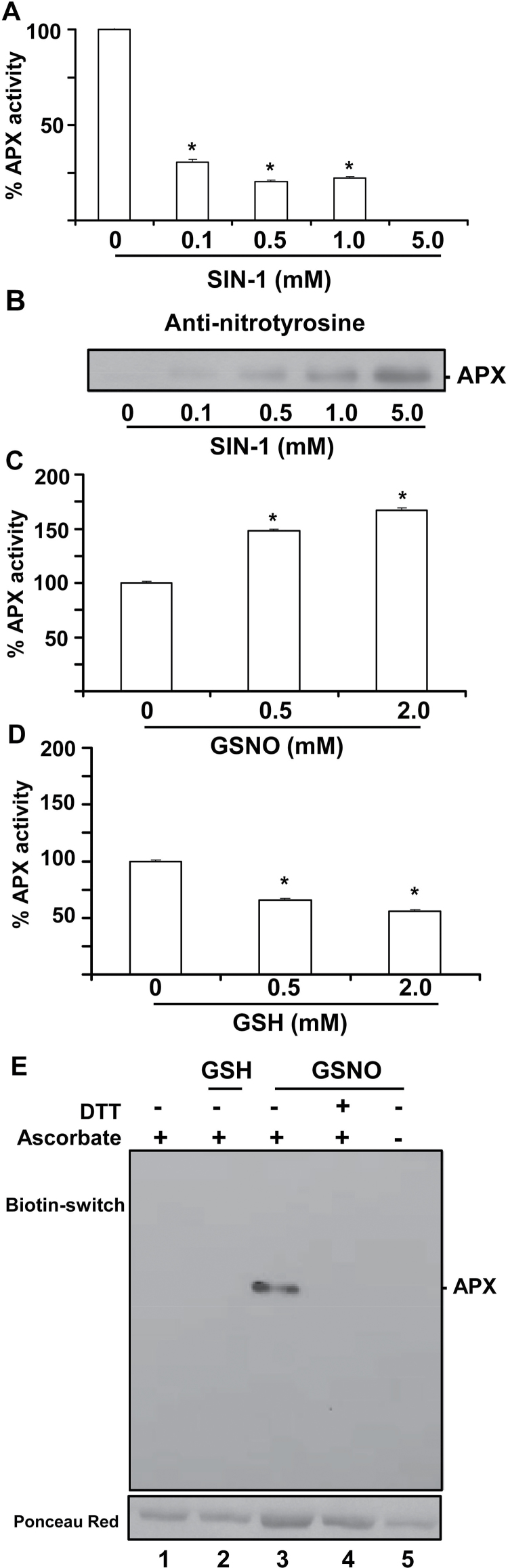
Effect of nitration (A and B) and *S*-nitrosylation on recombinant ascorbate peroxidase (APX) (C–E). (A) Effect of SIN-1 (peroxynitrite donor) on recombinant APX activity. (B) Representative immunoblot showing the grade of tyrosine nitration of APX treated with different concentrations of SIN-1 and detected with an antibody against 3-nitrotyrosine (dilution 1:2500). A 5 μg aliquot of protein was used per line. (C) Effect of *S*-nitrosoglutathione (GSNO). (D) Effect of glutathione (GSH). (E) *S*-nitrosylation of recombinant pea APX. A 5 μg aliquot of purified recombinant APX was treated with 2mM GSH and 2mM GSNO and was subjected to the biotin switch method. Control treatments were done with water (lane 1) and 2mM GSH (lane 2). Additionally, APX was *S*-nitrosylated with 2mM GSNO and reduced again with 50mM DTT (lane 4). Furthermore, GSNO-treated APX underwent the biotin switch method without ascorbate (lane 5). Proteins were separated under non-reducing conditions by SDS–PAGE and blotted onto a PVDF membrane. Biotinylated proteins were detected using anti-biotin antibodies. Ponceau red staining demonstrated equal loading.

In order to evaluate the potential action of different NO-derived molecules, an *in vitro* assay was carried out in the presence of ONOO^–^ using SIN-1 as the peroxynitrite donor (Chaki *et al.*, 2009) and GSNO as the NO donor. Figure 2A depicts the inhibitory effect of ONOO^–^ activity that ranges from 70% with 0.1mM SIN-1 to 100% with 5mM SIN-1. The reliability of the nitration by SIN-1 was confirmed by immunoblot analysis of the recombinant protein using an antibody against 3-nitrotyrosine. Figure 2B shows that the degree of nitration increases as a function of the SIN-1 concentration.

### Effect of *S*-nitrosylation of recombinant pea cytosolic APX

The effect of NO on APX is controversial. It has been reported to yield an increased APX activity in sweet potato (Lin *et al.*, 2011) and soybean (Keyster *et al.*, 2011), and an inhibition in tobacco (Clark *et al.*, 2000). Pea APX contains a single cysteine residue that is partially buried (with an ASA of 15 Å^2^) but that may be a good candidate for *S*-nitrosylation. In order to gain additional insight into the regulation of pea APX, the effect of increasing concentrations of GSNO, a well known NO donor, on the enzymatic activity was evaluated. As shown in Fig. 2C, 0.5mM and 2mM GSNO significantly increase the activity of APX. When activity was assayed in the presence of 0.5mM and 2mM GSH to evaluate whether this effect was due to the release and binding of NO to the protein, it was found that GSH yields a reduction in APX activity (Fig. 2D) which could be consequence of a process of *S*-glutathionylation (Dalle-Donne *et al.*, 2009). Since the enzymatic assay is based on measurement of the change of absorbance at 290nm as a consequence of the reduction of H_2_O_2_ with the concomitant oxidation of ascorbate, different combinations of GSNO, ascorbate, and H_2_O_2_ were assayed in the absence of the enzyme to rule out any possible interference in the assay. Supplementary Fig. S1 available at *JXB* online shows that none of these combinations yields significant variations in absorbance during a standard period of time used in the APX activity assay, confirming the reliability of the observed increase in activity of recombinant APX in the presence of GSNO.

Additionally, in order to confirm that GSNO treatment of recombinant APX undergoes a process of *S*-nitrosylation, the biotin switch method (Jaffrey *et al.*, 2001), which is specific for the detection of *S*-nitrosylated proteins, was assayed. Figure 2E shows that APX is *S*-nitrosylated after treatment with 2mM GSNO (lane 3), whereas the treatment with GSH does not produce any signal in the biotin switch assay (lane 2). Given the fact that the APX sequence has only one cysteine residue, Cys32 is the target of *S*-nitrosylation. As expected, *S*-nitrosylation of APX is reversible and it can be eliminated by adding a reducing agent such as DTT to the *S*-nitrosylated APX (lane 4) or in the absence of ascorbate (lane 5) which is used as an SNO-specific reducing agent, further demonstrating the *S*-nitrosylaton of Cys32.

### Characterization of nitrated recombinant pea cytosolic APX

**Fig. 3.**
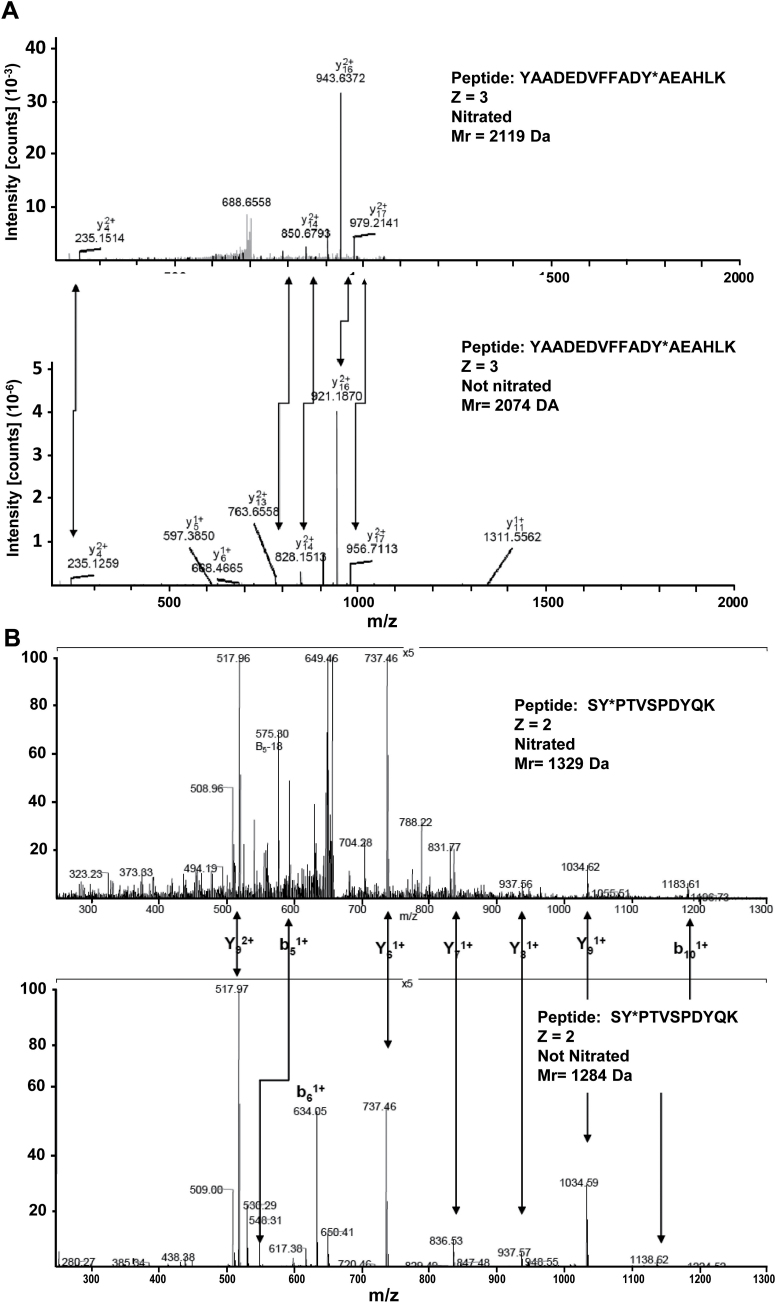
Comparison of the nitrated (top) and unmodified (bottom) MS/MS spectra of the identified peptides from pea APX in the corresponding panels: (A) YAADEDVFFADY*AEAHLK. (B) SY*PTVSPDYQK. Peptide fragment ions are indicated by ‘b’ if the charge is retained on the N-terminus and by ‘y’ if the charge is maintained on the C-terminus. The subscript indicates the number of amino acid residues in the considered fragment from either the N-terminus or the C-terminus. The superscript indicates the charge (1+ or 2+) of the backbone fragmentation.

With the aim of identifying which of the seven tyrosines present in the pea plant’s cytosolic APX is(are) a target(s) of this post-translational modification, peroxynitrite-treated recombinant APX was subjected to trypsin digestion followed by MALDI-TOF/TOF mass spectrometry examination. Table 1 shows the list of peptides scanned and those identified by LC-MS/MS. Among the peptides identified, only two contained a nitrated tyrosine. Figure 3 shows the comparison of the nitrated (top) and unmodified (bottom) MS/MS spectra of these identified peptides from the pea cytosolic APX. The nitrated peptide YAADEDVFFADYAEAHLK (Z=3) has a total of 18 amino acids and a mass of 2119Da (2074Da plus 45Da) which is compatible with the acquisition of a nitro group in Tyr235 (Fig. 3A). The nitrated peptide SYPTVSPDYQK (Z=2) has a total of 11 amino acids and a mass of 1329Da (1284Da plus 45Da) which is also compatible with the acquisition of a nitro group in Tyr5 (Fig. 3B).

### Analysis of the residues involved in the APX activity regulation by peroxynitrite and GSNO

Enzyme regulation plays an important role in homeostasis and it is reasonable to assume its preservation throughout evolution. Evolutionary analysis carried out at the Evolutionary Trace server (Mihalek *et al.*, 2004) using the tertiary structure of pea APX as input (PDB code 1apx) and the entire UniProtKB database (30,342,520 sequences) output rho values of 57.68 and 4.95 for Tyr5 and Tyr235, respectively, and 14.17 for Cys32 calculated from 416 sequences. Providing that the rho parameter deviates from 1 as the variability increases, Tyr5 is poorly preserved, Cys32 does not seem to be a critical residue, and only Tyr235 seems reasonably well preserved throughout evolution. However, when the analysis was focused on the 97 sequences with E-scores ranging from 508E-143 to 423E-117 found in the *Viridiplantae* subsection of UniProtKB (1,385,397 sequences), rho values were 1 for both Cys32 and Tyr235 and 1.79 for Tyr5. This result points to Cys32 and Tyr235 as evolutionarily relevant residues in plants, as expected from their role in the modulation of APX by *S*-nitrosylation and tyrosine nitration revealed by the present results. In this sense, Supplementary Fig. S2A at *JXB* online shows the multiple alignments of the amino acid sequence of pea cAPX with other 15 APX sequences of three model plants (*Arabidopsis thaliana*, *Medicago truncatula*, and *Lotus japonicus*) located in the different subcellular compartments including the cytosol, peroxisome, and chloroplast (stroma and thylakoid). Thus, it is remarkable that Tyr235 is absolutely conserved and Cys32 is present in all APXs except in APX03 and APX04 of *M. truncatula*. Additionally, Supplementary Fig. S2B shows the corresponding phylogenic tree where the APX sequences are divided into two categories: one comprising only MtAPx04 and another for the others. Interestingly, sequences are branched according to their subcellular location. The subtree for the peroxisomal APXs is subdivided into two branches: one for MtAPx03 and the other for the rest of the peroxisomal APXs. This analysis also suggests that both Tyr235 and Cys32 are important residues and that Tyr5 seems be a general feature of cytosolic APXs. Both MtAPx03 and MtAPx04 are exceptions, but the phylogenic tree shows that they are somehow peculiar: MtAPx04 is different from the others, regardless of the subcellular location, and MtAPx03, although being cytosolic, is also less similar.

**Fig. 4.**
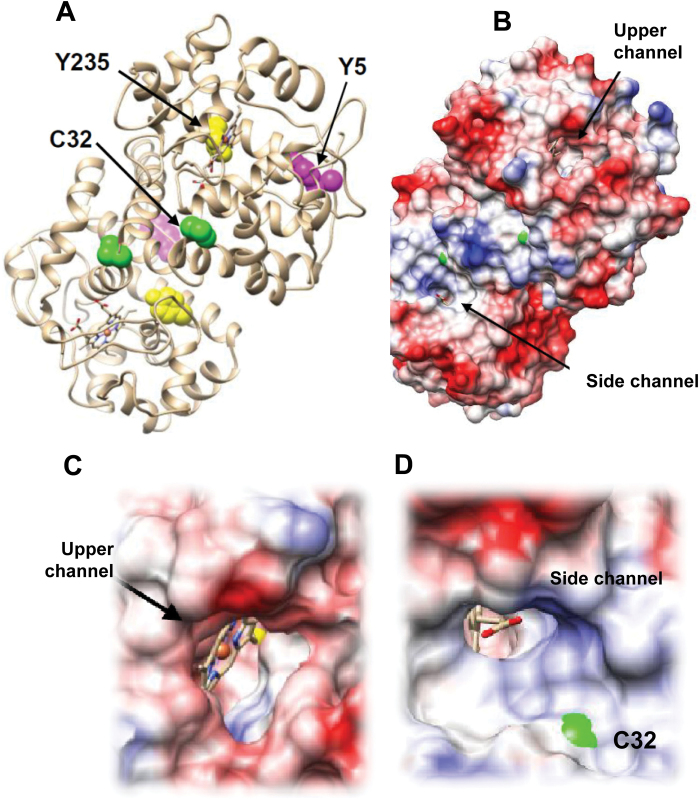
(A) Structure of homodimeric pea APX (PDB ID: 1apx). Residues identified as a target of tyrosine nitration and *S*-nitrosylation are shown as space filling. (B) The haem group is enclosed in a pocket with two channels to the exterior. (C) The view along the upper channel reveals that Y235 is at the bottom of the pocket. (D) C32 is located at the ascorbate binding site in the vicinity of the side channel. (This figure is available in colour at *JXB* online.)

The structure of pea APX has been solved at 2.2 Å resolution (Patterson and Poulos, 1995). It consists of a non-covalent homodimer with one haem group per monomer located in a pocket that it is opened to the exterior by two channels (Fig. 4A, B). A closer analysis shows that Tyr235 is located at the bottom of the pocket at 3.6 Å from the haem group (Fig. 4C) and Cys32 is close to the side channel (Fig. 4D). The location of Tyr5 does not reveal any functional role and since it is an accessible residue (ASA: 83 Å^2^) its nitration may not have a physiological relevance.

### Analysis of APX activity under salt-induced oxidative stress

**Fig. 5.**
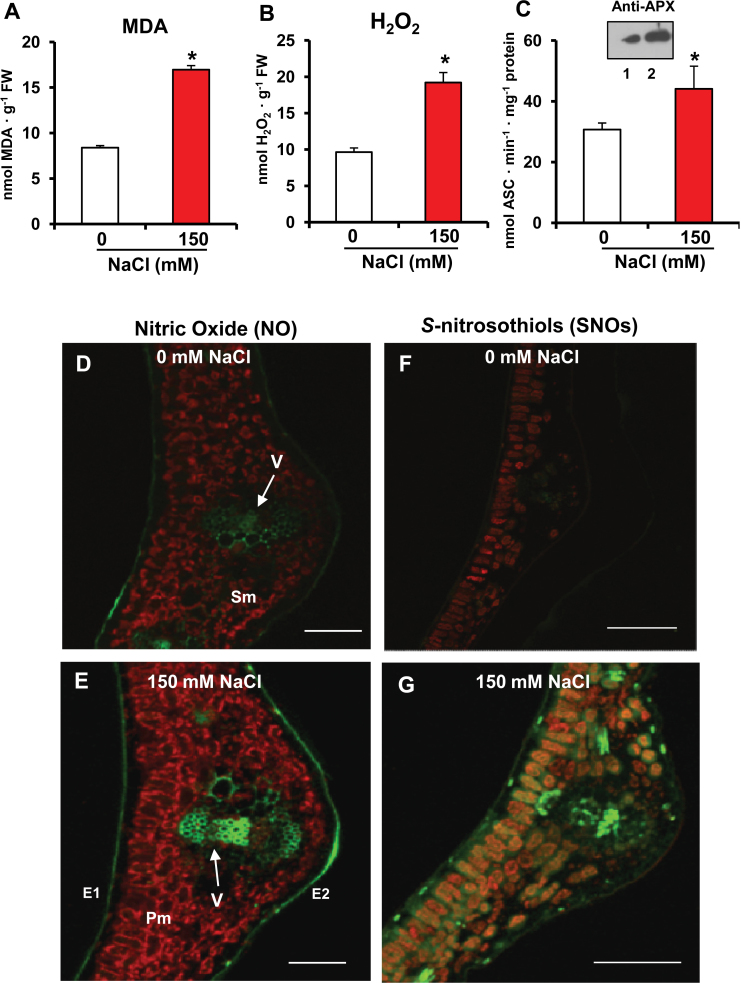
Lipid peroxidation, hydrogen peroxide, and ascorbate peroxidase (APX) activity, and representative images illustrating the CLSM detection and visualization of NO and *S*-nitrosothiols (SNOs) in leaves of pea plants exposed to 150mM NaCl. (A) Malondialdehyde (MDA) content. (B) Hydrogen peroxide content. (C) APX activity and western blotting analysis using an antibody against cucumber APX (dilution 1:3000). Lanes 1 and 2 correspond to leaf extracts of pea control plants and plants exposed to 150mM NaCl, respectively. (D) Detection of NO in a leaf cross-section of pea control plants (0mM NaCl). (E) Detection of NO in a leaf cross-section of pea plants exposed to 150mM NaCl. (F) Detection of SNOs in a leaf cross-section of pea control plants (0mM NaCl). (G) Detection of SNOs in a leaf cross-section of pea plants exposed to 150mM NaCl. NO and SNOs were detected with the fluorescent dyes DAF-FM DA and Alexa Fluor (AL) 488 Hg-link reagents, respectively, as described in the Materials and methods. The chlorophyll autofluorescence is shown. Adaxial epidermis (E1), abaxial epidermis (E2), main vein (V), palisade mesophyll (Pm), and spongy mesophyll (Sm). Bar=300 μm. Data are means ±SEM of at least three replicates. *Differences from control values were significant at *P* < 0.05. (This figure is available in colour at *JXB* online.)

In order to gain additional insight into the physiological relevance of APX activity under an oxidative stress situation, it was analysed in leaves of pea plants grown in the presence of 150mM NaCl. Special attention was paid to H_2_O_2_, NO, and SNO content since they may be interconnected with the activity of APX. As shown in Fig. 5, salt-induced stress yields a 12-fold increase of the content of MDA (Fig. 5A), a 2-fold increase in the content of H_2_O_2_ (Fig. 5B), and a 1.4-fold increase in the APX activity (Fig. 5C). By immunoblot, the APX protein expression was also evaluated, and it was found to increase under salinity conditions (Fig. 5C, lanes 1 and 2). The content and localization of NO and SNOs were analysed in leaf cross-sections by CLSM using DAF-FM DA and Alexa Fluor 488 Hg-link (Chaki *et al.*, 2009) as fluorescence probes, respectively. The results revealed a significant increase in both NO and SNO production, mainly in vascular tissue, in plants grown under saline stress (Fig. 5E, G) when compared with control plants (Fig. 5D, F).

### Purification of total *S*-nitrosylated proteins under salinity stress and detection of *S*-nitrosylated APX

**Fig. 6.**
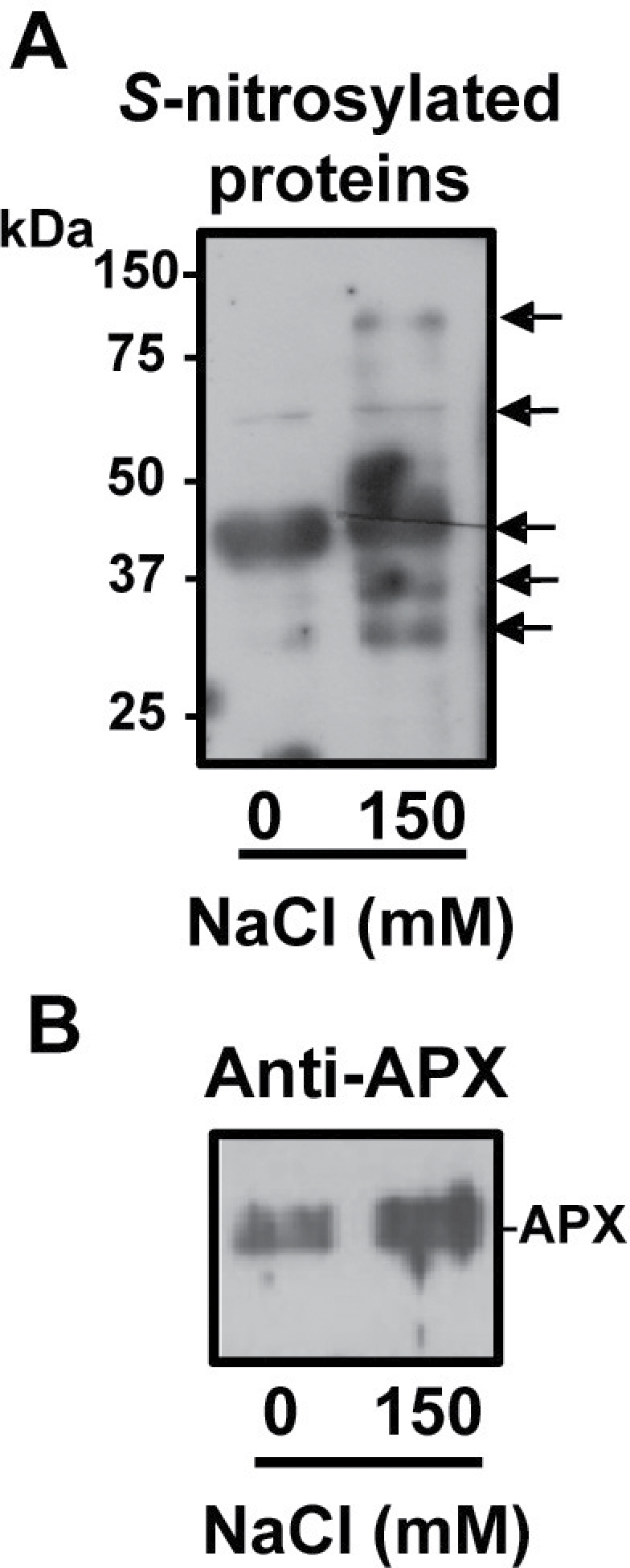
Total *S*-nitrosylated proteins and *S*-nitrosylated APX in leaves of pea plants under salinity stress conditions. (A) Detection of total *S*-nitrosylated proteins from leaves of pea plant controls and those exposed to 150mM NaCl. *S*-nitrosylated proteins were separated under non-reducing conditions by 12% SDS–PAGE and blotted onto a PVDF membrane. Biotinylated proteins were detected using anti-biotin antibodies as described in the Materials and methods. (B) Immunoblot of total *S*-nitrosylated proteins of leaves of pea plant controls and those exposed to 150mM NaCl probed with a polyclonal antibody against cucumber APX (dilution 1:3,000). A 5 μg aliquot of protein was used per lane.

To evaluate if APX under salinity stress conditions undergoes a process of *S*-nitrosylation, total *S*-nitrosylated proteins were purified from leaves of pea plants grown under control and salinity stress conditions and then the presence of APX protein among these *S*-nitrosylated protein was evaluated by immunoblots. Figure 6A depicts the electrophoretic analysis of total *S*-nitrosylated proteins. Thus, under salinity stress, the pattern of *S*-nitrosylated proteins showed an increase in the number and in the intensity of some specific bands. Figure 6B shows the immunoblot analysis of the total *S*-nitrosylated proteins probed with an antibody against cucumber APX where an increase under salinity stress was observed. Taken together, the results indicate that APX is *S*-nitrosylated *in vivo* and this process is increased under salinity conditions. which supports the data observed in *in vitro* conditions

## Discussion

APX is one of the key antioxidant enzymes involved in the regulation of H_2_O_2_ levels during plant development and under adverse stress conditions (Jiménez *et al.*, 1998; Gomez *et al.*, 2004; Palma *et al.*, 2006; Leterrier *et al.*, 2007). Given that independent proteomic studies have identified APX as a potential target of both nitration (Lozano-Juste *et al.*, 2011) and *S*-nitrosylation (Bai *et al.*, 2011; Fares *et al.*, 2011), a pharmacological study using recombinant pea cytosolic APX was undertaken to identify which amino acid residue(s) is(are) potential target(s) of these PTMs and the effect(s) on APX activity.

In higher plants, proteomic analyses of nitration have shown that a certain number of proteins are targets of this PTM mediated by NO-derived molecules. However, information on the specific impact on particular proteins involved in antioxidative systems and on the consequence of tyrosine nitration on activity and protein structure is scarce (Radi, 2013). Up to now, most analyses have shown that nitration usually causes a loss of function (Corpas *et al.*, 2013). Activity loss in the case of APX thus concurs with previous observations of tobacco APX (Clark *et al.*, 2000) and those of other protein activities such as ferredoxin-NADP reductase, carbonic anhydrase (CA), *S*-adenosyl homocysteine hydrolase (Chaki *et al*., 2012, 2013*b*), *O*-acetylserine(thiol)lyase A1 (Álvarez *et al.*, 2011), and NADP-isocitrate dehydrogenase (Begara-Morales *et al.*, 2013*a*). In this study, mass spectrometry analysis shows that two tyrosines are targets of nitration. However, of the two nitrated tyrosines, Tyr235 is the most reliable candidate to provoke the observed inhibition of the APX activity since this residue is at the bottom of the pocket where the catalytic centre is located, only 3.6 Å away from the haem group (Patterson and Poulos, 1995; Jespersen *et al.*, 1997; Mandelman *et al.*, 1998). The addition of the nitro group may disrupt the properties of the haem group to result in a loss of the activity. However, the existence of two kinetically competent binding sites for ascorbate has been reported (Mandelman *et al.*, 1998) and the facts that Tyr5 is relatively well preserved in plants (rho value 1.79) and that Tyr5 is either present or absent, but never replaced by other residues, seem to point to a physiological role for its nitration, although its location makes it difficult to rationalize the consequences on the enzymatic activity.

In higher plants, the biotin switch method has become a reliable way of detecting protein target candidates for *S*-nitrosylation (Sell *et al.*, 2008). Consequently, the number of identified proteins affected by this process using the biotin switch method continues to grow. However, the specific regulatory impact is largely unknown in the majority of cases (Astier *et al.*, 2012; Lin *et al.*, 2012; Tanou *et al.*, 2012), and only in a limited number of cases have their affects been clearly identified. In *Arabidopsis thaliana,* for example, cytosolic glyceraldehyde-3-phosphate dehydrogenase undergoes a reversible inhibition by NO (Lindermayr *et al.*, 2005) and catalytic Cys155 and Cys159 appear to be targets for *S*-nitrosylation (Holtgrefe *et al.*, 2008). More recently, it has been reported that plant NADPH oxidase (AtRBOHD) is a target of *S*-nitrosylation at Cys890 *in vitro* and also *in vivo* during *Pseudomonas syringae* infection. Cys890 is situated close to Phe921 which is involved in the FAD binding site (Yun *et al.*, 2011). The impact is known in other cases such as methionine adenosyltransferase which is inhibited by *S*-nitrosylation (Lindermayr *et al.*, 2006) and *Arabidopsis* type-II metacaspase AtMC9 which blocks the autoprocessing and activation of AtMC9 zymogen through *S*-nitrosylation of its catalytic cysteine residue (Belenghi *et al.*, 2007).

Using proteomic studies, APX has been identified as a potential target of *S*-nitrosylation (Fares *et al.*, 2011). A previous work described the inhibition of APX activity by increasing concentrations of GSNO in tobacco leaf extracts (Clark *et al.*, 2000). However more recent results showed the opposite behaviour, with enhanced activity of different APX isozymes in root nodules of soybean treated with an NO donor (Keyster *et al.*, 2011). Similarly, in seeds of *Anticaria toxicaria* treated with NO gas, the *S*-nitrosylation of APX was described, which also enhanced its activity which it seemed to contribute during seed desiccation (Bai *et al.*, 2011). The present results showing that GSNO enhances the activity of pea APX and *S*-nitrosylation is corroborated by the biotin switch method, with Cys32 being the target of the *S*-nitrosylation. Cys32 is near the propionate side chain of the haem group and it has been reported to form thiyl radicals through interaction of APX with H_2_O_2_ (Kitajima *et al.*, 2008), supporting a direct reaction with NO (Martinez-Ruiz and Lamas, 2007). However, the mechanism underlying the activation by *S*-nitrosylation is far from trivial since Cys32 does not seem to be a critical residue in the catalytic process. In fact, mutation of Cys32 only provokes a 3-fold decrease on the ascorbate peroxidase activity (Mandelman *et al.*, 1998), and the structure of the soybean APX has revealed that Cys32 has no direct interaction with ascorbate, suggesting that the 1000-fold inhibition caused by DNTB [5,5′-dithiobis-(2-nitrobenzoic acid)] is due to the blockage of the side channel (Sharp *et al.*, 2003). It has also been reported that cysteine oxidation provokes loss of APX-B enzyme activity although the reason for this is not clear. Interestingly, the oxidation of Cys32 causes enzyme inactivation, and it has been suggested that glutathionylation protects the enzyme from irreversible oxidation (Kitajima *et al.*, 2008). In this context, one might hypothesize that *S*-nitrosylation prevents APX from inactivation by H_2_O_2_ to yield an increase of the activity when compared with unprotected enzyme.

Very recently, proteomic analysis of *Arabidopsis* roots identified the cytosolic APX (APX1) as a target of *S*-nitrosylation, and *in vitro S*-nitrosylation of the recombinant APX1 showed that this process provoked an increase in its activity (Correa-Aragunde *et al.*, 2013). However, the authors, using an *in silico* analysis, proposed that among the five cysteine residues present in the *Arabidopsis* APX1, Cys168 could be the target of *S*-nitrosylation.

In order to evaluate the physiological role of APX activity under stress conditions, salinity stress was selected because it has been reported to yield both oxidative and nitrosative stress (Hernández *et al.*, 1995; Gómez *et al.*, 2004; Corpas *et al.*, 2009; Leterrier *et al.*, 2012; Tanou *et al.*, 2012). Concomitant with an enhancement of the activity of APX, key elements in the metabolism of ROS and RNS, including lipid peroxidation, H_2_O_2_, NO, and SNOs, are significantly increased under the saline stress induced by 150mM NaCl. The fact that *S*-nitrosylation of Cys32 causes an increase in APX activity may suggest that this PTM might be involved in the specific case of salinity stress which is accompanied by both oxidative stress and a rise in SNOs.

In summary, the present results provide new insights into the dual mechanism of regulation of APX by post-translational modification mediated by NO-derived molecules. It is interesting to note that these NO-related PTMs produce opposite effects on the enzymatic activity, with a different consequence on the long-term functionality of the proteins, since the modulation by *S*-nitrosylation is reversible whereas tyrosine nitration leads to an irreversible inhibition of the enzyme. To the authors’ knowledge, these two PTMs have not been described in relation to plant APX which is a key element in the fine-tuning regulation of H_2_O_2_ and consequently in the mechanism of signalling and response during plant development and/or against adverse stress conditions such as salinity. Moreover, it is shown that *S*-nitrosylation occurs in vivo and that this PTM is accentuated under salinity conditions as a consequence of an increase in both NO and SNOs. In these circumstances, a rise of APX activity is observed, which, in part, is due to a process of *S*-nitrosylation. This suggests that SNOs contribute to alleviate oxidative damage induced by salinity stress. Thus, the results also support the existence of an additional interplay between the metabolism of ROS and RNS in higher plants, which is in agreement with some previous data where the metabolism of both ROS and RNS under specific environmental stress conditions can cause oxidative as well as nitrosative stress (Valderrama *et al.*, 2007; Molassiotis and Fotopoulos, 2011; Corpas and Barroso, 2013).

## Supplementary data

Supplementary data are available at JXB online.

Figure S1. Control of the interaction effects on *A*_290_ of ascorbate, GSNO, and H_2_O_2_.

Figure S2. Multiple sequence alignments (A) and phylogenic tree (B) of the amino acid sequence of pea cAPX and other 15 APX sequences.

Supplementary Data

## Abbreviations:

NO: nitric oxide
NO_2_-Tyr: 3-nitrotyrosine
PTM: post-translational modification
ROS: reactive oxygen species
RNS: reactive nitrogen species.
