# Supplementary material for: Dual regulation of cytosolic ascorbate peroxidase (APX) by tyrosine nitration and S-nitrosylation
Source: J Exp Bot. 2013 Nov 28;65(2):527–38. doi: 10.1093/jxb/ert396 (PMC3904709; doi:10.1093/jxb/ert396)
Supplement: Supplementary Data [file supp_65_2_527__index.html]

Dual regulation of cytosolic ascorbate peroxidase (APX) by tyrosine nitration and S-nitrosylation — Dual regulation of cytosolic ascorbate peroxidase (APX) by tyrosine nitration and S-nitrosylation — Supplementary Data 

# Dual regulation of cytosolic ascorbate peroxidase (APX) by tyrosine nitration and *S*-nitrosylation

## Supplementary Data

Data files

**Files in this Data Supplement:**

- Supplementary Data - Supplementary Data
