# Supplementary material for: Dual regulation of cytosolic ascorbate peroxidase (APX) by tyrosine nitration and S-nitrosylation
Source: J Exp Bot. 2013 Nov 28;65(2):527–38. doi: 10.1093/jxb/ert396 (PMC3904709; doi:10.1093/jxb/ert396)
Supplement: Supplementary Data [file supp_ert396_jexbot106138_file001.pdf]

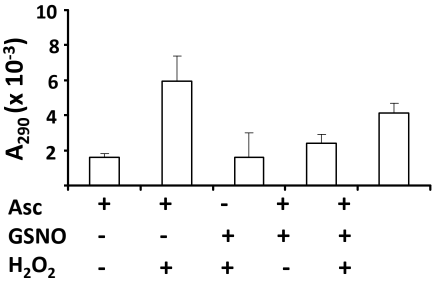


**Supplemental Figue 1.** Interaction effects on $A_{290}$ of between ascorbate , GSNO and $H_2O_2$. Purified APX protein was incubated at different concentrations of SIN-1 at 37ºC for 60 min, GSNO at 25ºC for 30 min or GSH at 25ºC for 30 min. The specific activity of the recombinant APX was 204 nmol ascorbate · $min^{-1}$ · $mg^{-1}$ protein. Data are means ± SEM of at least three replicates. *Differences from control values were significant at $P < 0.05$.

**Supplemental Figure 2**

**Multiple sequence alignments(A) and phylogenic tree (B) of the amino acid sequence of pea cAPX and other 15 APX sequences found in Peroxibase (http://peroxibase.toulouse.inra.fr/) from the three model plants *Arabidopsis thaliana*, *Medicago truncatula* and *Lotus japonicus* with different subcellular localization including cytosol, peroxisome and chloroplast (stroma and thylakoid).** Six APXs are from *A. thaliana*, including two cytosolic (AtAPx01, AtAPx02)**,** 2 peroxisomal (AtAPx03, AtAPx04)**,** 1 stromal (AtAPx05)**,** 1 thylakoid-bound (AtAPx06) and 1 thylakoid-lumen that is remarked as not as APX (AtAPx07)**;** seven APXs from *M. truncatula*: 2 cytosolic **(**MtAPx01, MtAPx03)**,** 2 peroxisomal **(**MtAPx02, MtAPx05)**,** 2 thylakoid-bound **(**MtAPx07-1, MtAPx07-2) and 1 un-localized **(**MtAPx05); and two APXs from *L. japonicus*: peroxisomal (LjAPx02) and cytosolic (LjAPx01). The relative position of Tyr-5, Tyr-235 and Cys-32 of pea cAPX is indicated in the protein sequences by yellow color. Single asterisk indicates identical residues. Semi-colon indicates conservative substitutions. Period indicate less conservative substitutions.

**(A)**

```
MtAPx07-1_thylakoid-bound      -----------------MATITGGAAARMIPSATRATVSLSTSRSFFSFS
MtAPx07-2_thylakoid-bound      -----------------MATITGGAAARMIPSATRATVSLSTSRSFFSFS
AtAPx06_thylakoid-bound        -----------------MSVSLSAASHLLCSSTRVSLSPAVTSSSSSPVV
AtAPx05_stromal                MAERVSLTLNGTLLSPPPTTTTTTMSSSLRSTTAASLLLRSSSSSSRSTL
LjAPx02_peroxisomal            --------------------------------------------------
MtAPx02_peroxisomal            --------------------------------------------------
AtAPx03_peroxisomal            --------------------------------------------------
MtAPx05_peroxisomal            --------------------------------------------------
AtAPx04_peroxisomal            --------------------------------------------------
PsAPX_cytosolic                --------------------------------------------------
MtAPx01_cytosolic              --------------------------------------------------
LjAPx01_cytosolic              --------------------------------------------------
MtAPx03_cytosolic              --------------------------------------------------
AtAPx01_cytosolic              --------------------------------------------------
AtAPx02_cytosolic              --------------------------------------------------
MtAPx04_N/D                    -------MGVSFLSISTLPSLHPLGGSHYPKTTSTRHQPRSVSICCAKIK

MtAPx07-1_thylakoid-bound      LASSSRSVSSLNCLRSSPRISHIFLNQRRGEVR------VSSGRFGTVAF
MtAPx07-2_thylakoid-bound      LASSSRSVSSLNCLRSSPRISHIFLNQRRGEVR------VSSGRFGTVAF
AtAPx06_thylakoid-bound        ALSSSTSPHSLGSVASSSLFPHSSFVLQKKHPIN----GTSTRMISPKCA
AtAPx05_stromal                TLSASSSLSFVRSLVSSPRLSSSSSLSQKKYRIASVNRSFNSTTAATKSS
LjAPx02_peroxisomal            ------------------------------------------MALPVVVD
MtAPx02_peroxisomal            ------------------------------------------MAFPVVVD
AtAPx03_peroxisomal            -------------------------------------------MAAPIVD
MtAPx05_peroxisomal            -------------------------------------------MAKYAVD
AtAPx04_peroxisomal            --------------------------------------------MAVNVD
PsAPX_cytosolic                ------------------------------------------GKSYPTVS
MtAPx01_cytosolic              -----------------------------------------MGKSYPTVS
LjAPx01_cytosolic              -----------------------------------------MGKSYPTVS
MtAPx03_cytosolic              -------------------------------------------------M
AtAPx01_cytosolic              -----------------------------------------MTKNYPTVS
AtAPx02_cytosolic              ----------------------------------------MVKKSYPEVK
MtAPx04_N/D                    TDVSDEEEQFFHWRRRDVLKCIGLTIGLESISSSGSLLGTANAADLIERR

MtAPx07-1_thylakoid-bound      ASDPDQLKSAREDIKELLKTKFCHPLLIRLGWHDAGTYNKNIEEWPQRGG
MtAPx07-2_thylakoid-bound      ASDPDQLKSAREDIKELLKTKFCHPLLIRLGWHDAGTYNKNIEEWPQRGG
AtAPx06_thylakoid-bound        ASDAAQLISAKEDIKVLLRTKFCHPILVRLGWHDAGTYNKNIEEWPLRGG
AtAPx05_stromal                SSDPDQLKNAREDIKELLSTKFCHPILVRLGWHDAGTYNKNIKEWPQRGG
LjAPx02_peroxisomal            GDYLKEVDKARRDLRALIANRNCAPLMLRLAWHDAGTYDA----RTKTGG
MtAPx02_peroxisomal            SEYLKEIEKARRDLRALIANRNCAPLMLRLAWHDAGTYDA----KTKTGG
AtAPx03_peroxisomal            AEYLKEITKARRELRSLIANKNCAPIMLRLAWHDAGTYDA----QSKTGG
MtAPx05_peroxisomal            AEYVKEIEEVRRDLRSFITNKKCAPLMLRLAWHDAGTYDA----KTRTGG
AtAPx04_peroxisomal            AEYLKEIEKTRRDLRALISSRNCAPIMLRLAWHDAGTYDA----KKKTGG
PsAPX_cytosolic                PDYQKAIEKAKRKLRGFIAEKKCAPLILRLAWHSAGTFDS----KTKTGG
MtAPx01_cytosolic              ADYQKAVEKAKRKLRGFIAEKKCAPLMLRLAWHSAGTFDS----KTKTGG
LjAPx01_cytosolic              ADYQKAIEKARRKLRGFIADKKCAPLMLRLAWHSAGTFDV----KTKTGG
```

```
MtAPx03_cytosolic              QTQTPALNASRKKLVELIETSRNVWLIFHWKS------------------
AtAPx01_cytosolic              EDYKKAVEKCRRKLRGLIAEKNCAPIMVRLAWHSAGTFDC----QSRTGG
AtAPx02_cytosolic              EEYKKAVQRCKRKLRGLIAEKHCAPIVLRLAWHSAGTFDV----KTKTGG
MtAPx04_N/D                    QRSDFQSQIKGTLYTAIKANPDLTPSILTLALNDALTYDK----ATKTGG
                                               :         :.

MtAPx07-1_thylakoid-bound      ANGSLRFEVELKHGANAGLVNALKLLQPIKDKYSGVTYADLFQLASATAV
MtAPx07-2_thylakoid-bound      ANGSLRFEVELKHGANAGLVNALKLLQPIKDKYSGVTYADLFQLASATAV
AtAPx06_thylakoid-bound        ANGSLRFEAELKHAANAGLLNALKLIQPLKDKYPNISYADLFQLASATAI
AtAPx05_stromal                ANGSLRFDIELKHAANAGLVNALNLIKDIKEKYSGISYADLFQLASATAI
LjAPx02_peroxisomal            PNGSIRNEEEFSHGANNGLKKALDFCEEVKTKVPKITYADLYQLAGVVAV
MtAPx02_peroxisomal            PNASIRNEEEFSHGANNGLKKAIDFCEEVKAKHPKISYADLYQLAGVVAV
AtAPx03_peroxisomal            PNGSIRNEEEHTHGANSGLKIALDLCEGVKAKHPKITYADLYQLAGVVAV
MtAPx05_peroxisomal            PNGSIRNQQELNHAANKGLKTAVELCEEVKVKHPKVSYADLYQLAGVVAV
AtAPx04_peroxisomal            ANGSIRFKEELNRPHNKGLEKAVAFCEEVKAKHPRVSYADLYQLAGVVAV
PsAPX_cytosolic                PFGTIKHQAELAHGANNGLDIAVRLLEPIKEQFPIVSYADFYQLAGVVAV
MtAPx01_cytosolic              PFGTIKHQAELAHGANNGLDIAVRLLEPLKEQFPIISYADFYQLAGVVAV
LjAPx01_cytosolic              PFGTMKHAAELAHGANNGLDIAVRLLEPLKEQFPILSYADFYQLAGVVAV
MtAPx03_cytosolic              ----------------------KVILEPLKEQFLIISYVDFYQLSGVVAV
AtAPx01_cytosolic              PFGTMRFDAEQAHGANSGIHIALRLLDPIREQFPTISFADFHQLAGVVAV
AtAPx02_cytosolic              PFGTIRHPQELAHDANNGLDIAVRLLDPIKELFPILSYADFYQLAGVVAV
MtAPx04_N/D                    PNGSIRFSSEISRPENKGLSAALNFIEEAKKEIDSYSKGGPISYADLIQY
                                                       : .  :      :  .  . :.

MtAPx07-1_thylakoid-bound      EEAGGPKIPMKYGRVDVTGPEQCPEEGRLPDAGPPSPADHLRQVFY-RMG
MtAPx07-2_thylakoid-bound      EEAGGPKIPMKYGRVDVTGPEQCPEEGRLPDAGPPSPADHLRQVFY-RMG
AtAPx06_thylakoid-bound        EEAGGPDIPMKYGRVDVVAPEQCPEEGRLPDAGPPSPADHLRDVFY-RMG
AtAPx05_stromal                EEAGGPKIPMKYGRVDASGPEDCPEEGRLPDAGPPSPATHLREVFY-RMG
LjAPx02_peroxisomal            EITGGPTINFVPGRKDS---KISTNEGRLPDA--KQGVPHLRDIFY-RMG
MtAPx02_peroxisomal            EVTGGPTVNFVPGRRDS---KVCTRDGRLPDA--KQGVSHLRDIFY-RMG
AtAPx03_peroxisomal            EVTGGPDIVFVPGRKDS---NVCPKEGRLPDA--KQGFQHLRDVFY-RMG
MtAPx05_peroxisomal            EVTGGPTIQFVPGRKDS---LESPQEGRLPDA--KQGASHLREIFY-RMG
AtAPx04_peroxisomal            EVTGGPAIPFTPGRKD----ADSADDGELPNP--NEGASHLRTLFS-RMG
PsAPX_cytosolic                EITGGPEVPFHPGREDK---PEPPPEGRLPDA--TKGSDHLRDVFGKAMG
MtAPx01_cytosolic              EVTGGPEVPFHPGREDK---PEPPPEGRLPDA--TKGSDHLRDVFGKAMG
LjAPx01_cytosolic              EVTGGPDVPFHPGREDK---PQPPPEGRLPDA--TKGSDHLRDVFVKTMG
MtAPx03_cytosolic              EITGGPEVPFHPGGEDK---PEPPLEGRLPDA--TEGSNHLRDVFGKSMG
AtAPx01_cytosolic              EVTGGPDIPFHPGREDK---PQPPPEGRLPDA--TKGCDHLRDVFAKQMG
AtAPx02_cytosolic              EITGGPEIPFHPGRLDK---VEPPPEGRLPQA--TKGVDHLRDVFG-RMG
MtAPx04_N/D                    AAQSATKAQFLASAIRK--CGGNEEKGNLLYT--AYGSNGQWGLFDRQFG
                                  ...   :  .            .*.*  .          :*   :*

MtAPx07-1_thylakoid-bound      LNDKEIVALSGAHTLGRSRPDRSGWGKPETKYTKDGPGAPGGQSWTAQWL
MtAPx07-2_thylakoid-bound      LNDKEIVALSGAHTLGRSRPDRSGWGKPETKYTKDGPGAPGGQSWTAQWL
AtAPx06_thylakoid-bound        LDDKEIVALSGAHTLGRARPDRSGWGKPETKYTKTGPGEAGGQSWTVKWL
AtAPx05_stromal                LDDKDIVALSGAHTLGRSRPERSGWGKPETKYTKEGPGAPGGQSWTPEWL
LjAPx02_peroxisomal            LTDKDIVALSGAHTLGRAHPERSGFDGP----------------WTEDPL
MtAPx02_peroxisomal            LTDKDIVALSGAHTLGRAHPERSGFDGP----------------WTEDPL
AtAPx03_peroxisomal            LSDKDIVALSGGHTLGRAHPERSGFDGP----------------WTQEPL
MtAPx05_peroxisomal            LTDKDIVALSGGHTLGKAHKDRSNFEGQ----------------WTRDPL
AtAPx04_peroxisomal            LLDRDIVALSGGHTLGRAHKERSDFEGP----------------WTQDPL
PsAPX_cytosolic                LSDQDIVALSGGHTIGAAHKERSGFEGP----------------WTSNPL
MtAPx01_cytosolic              LSDQDIVALSGGHTIGAAHKERSGFEGP----------------WTSNPL
LjAPx01_cytosolic              LNDVDIVALSGGHTLGAAHKERSGFEGP----------------WTTNPH
MtAPx03_cytosolic              LSDQDIVALSGXHTIGAAHKERSGFEGP----------------WTSNPL
AtAPx01_cytosolic              LSDKDIVALSGAHTLGRCHKDRSGFEGA----------------WTSNPL
AtAPx02_cytosolic              LNDKDIVALSGGHTLGRCHKERSGFEGA----------------WTPNPL
MtAPx04_N/D                    RTDTDEADPEGRIPLWEKASVQEMKDKFS----------------AVGFG
                                 * : .  .*  .:      :.                      :

MtAPx07-1_thylakoid-bound      KFDNSYFKDIKEKKDEDLLVLPTDAALFDDPSFKVYAEKYAVDQEAFFKD
MtAPx07-2_thylakoid-bound      KFDNSYFKDIKEKKDEDLLVLPTDAALFDDPSFKVYAEKYAVDQEAFFKD
AtAPx06_thylakoid-bound        KFDNSYFKDIKEKRDDDLLVLPTDAALFEDPSFKNYAEKYAEDVAAFFKD
AtAPx05_stromal                KFDNSYFKEIKEKRDEDLLVLPTDAAIFEDSSFKVYAEKYAADQDAFFKD
LjAPx02_peroxisomal            KFDNSYFVELLKEESAGLLKLPTDKALLEDPAFRRYVELYAKDEEAFFRD
MtAPx02_peroxisomal            KFDNSYFQILLEEDSAALLKLPTDRALLDDPEFRRYVELYAKDEDAFFRD
AtAPx03_peroxisomal            KFDNSYFVELLKGESEGLLKLPTDKTLLEDPEFRRLVELYAKDEDAFFRD
MtAPx05_peroxisomal            KFDNSYFVELMKLESNDLLKLPTDKALVDDPAFRKYVELYAKDENAFFRD
AtAPx04_peroxisomal            KFDNSYFVELLKGETPGLLQLKTDKALLDDPKFHPFVKLYAKDEDMFFKA
PsAPX_cytosolic                IFDNSYFTELLTGEKDGLLQLPSDKALLTDSVFRPLVEKYAADEDVFFAD
MtAPx01_cytosolic              IFDNSYFTELLGGEKEGLLQLPSDKALLSDPVFRPLVEKYAADEDAFFAD
```

```
LjAPx01_cytosolic              IFDNTYFTELLTGEKEGLLQLPTDKALLSDPVFRPLVDKYAADEDAFFAD
MtAPx03_cytosolic              IFDNSYFTKLLGGEKEGLLQLPSDKALLSDLVFRLLVEKYVADEDAFFAD
AtAPx01_cytosolic              IFDNSYFKELLSGEKEGLLQLVSDKALLDDPVFRPLVEKYAADEDAFFAD
AtAPx02_cytosolic              IFDNSYFKEILSGEKEGLLQLPTDKALLDDPLFLPFVEKYAADEDAFFED
MtAPx04_N/D                    PRQLAVLSAFIGPDQDATETLLASDPDVAPWVQKYQRSRETVSRTDYEVD
                                 : : :  :          * :. . .         .  . .   :

MtAPx07-1_thylakoid-bound      YAEAHAKLSNLGAKFEPAEGVVVDGSPNVVGEKFVAAKYSSGK-------
MtAPx07-2_thylakoid-bound      YAEAHAKLSNLGAKFEPAEGVVVDGSPNVVGEKFVAAKYSSGKKELSDAM
AtAPx06_thylakoid-bound        YAEAHAKLSNLGAKFDPPEGIVIEN----VPEKFVAAKYSTGKKELSDSM
AtAPx05_stromal                YAVAHAKLSNLGAEFNPPEGIVI---------------------------
LjAPx02_peroxisomal            YAESHKKLSELGFVP-SSKASSPKDGTILLQSAVGVVVTGAVVILSYLYE
MtAPx02_peroxisomal            YAESHKKLSELGFAP-SSKATAPVDATVLAQSAFGVVAAAAVVILGYLYE
AtAPx03_peroxisomal            YAESHKKLSELGFNPNSSAGKAVADSTILAQSAFGVAVAAAVVAFGYFYE
MtAPx05_peroxisomal            YATSHKKLSELGFNP------SCSYRSQLAKAALGMVIASTVVVLGYLLE
AtAPx04_peroxisomal            YAISHKKLSELGFNP------PRRIPSAVTQQTLGIAVAAAVVIFTICYE
PsAPX_cytosolic                YAEAHLKLSELGFAEA----------------------------------
MtAPx01_cytosolic              YAEAHQKLSELGFAEA----------------------------------
LjAPx01_cytosolic              YAESHRKLSELGFADA----------------------------------
MtAPx03_cytosolic              YVEARQKLFELGFAEA----------------------------------
AtAPx01_cytosolic              YAEAHMKLSELGFADA----------------------------------
AtAPx02_cytosolic              YTEAHLKLSELGFADKE---------------------------------
MtAPx04_N/D                    LITTFTKLSTLGQKINYEAYTYPRKKIDITKLKL----------------
                                  :  **  **

MtAPx07-1_thylakoid-bound      --------------------------------------------------
MtAPx07-2_thylakoid-bound      RKKIRAEYEAVGGSPDKALKSNYFLNIIIVIAALAILTYLFGN-------
AtAPx06_thylakoid-bound        KKKIRAEYEAIGGSPDKPLPTNYFLNIIIAIGVLVLLSTLFGGNNNSDFS
AtAPx05_stromal                --------------------------------------------------
LjAPx02_peroxisomal            IRKRAK--------------------------------------------
MtAPx02_peroxisomal            THKRGK--------------------------------------------
AtAPx03_peroxisomal            IRKRMK--------------------------------------------
MtAPx05_peroxisomal            LNKKIN--------------------------------------------
AtAPx04_peroxisomal            ASRRGK--------------------------------------------
PsAPX_cytosolic                --------------------------------------------------
MtAPx01_cytosolic              --------------------------------------------------
LjAPx01_cytosolic              --------------------------------------------------
MtAPx03_cytosolic              --------------------------------------------------
AtAPx01_cytosolic              --------------------------------------------------
AtAPx02_cytosolic              --------------------------------------------------
MtAPx04_N/D                    --------------------------------------------------


MtAPx07-1_thylakoid-bound      --
MtAPx07-2_thylakoid-bound      --
AtAPx06_thylakoid-bound        GF
AtAPx05_stromal                --
LjAPx02_peroxisomal            --
MtAPx02_peroxisomal            --
AtAPx03_peroxisomal            --
MtAPx05_peroxisomal            --
AtAPx04_peroxisomal            --
PsAPX_cytosolic                --
MtAPx01_cytosolic              --
LjAPx01_cytosolic              --
MtAPx03_cytosolic              --
AtAPx01_cytosolic              --
AtAPx02_cytosolic              --
MtAPx04_N/D                    --
```

**(B)**

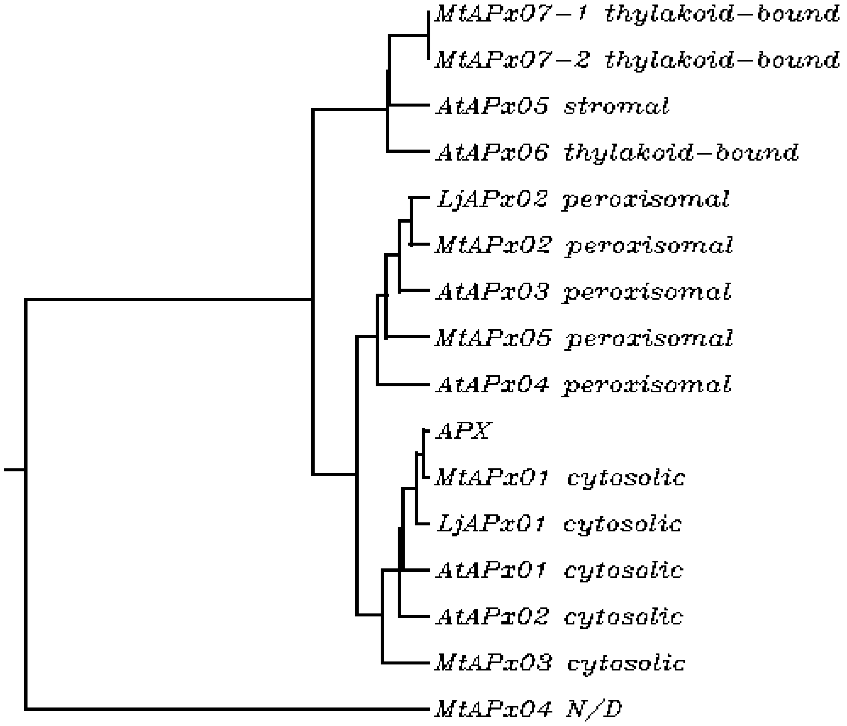
MtAPx07−1 thylakoid−bound
MtAPx07−2 thylakoid−bound
AtAPx05 stromal
AtAPx06 thylakoid−bound
LjAPx02 peroxisomal
MtAPx02 peroxisomal
AtAPx03 peroxisomal
MtAPx05 peroxisomal
AtAPx04 peroxisomal
APX
MtAPx01 cytosolic
LjAPx01 cytosolic
AtAPx01 cytosolic
AtAPx02 cytosolic
MtAPx03 cytosolic
MtAPx04 N/D
